# Albumin-based cancer therapeutics for intraperitoneal drug delivery: a review

**DOI:** 10.1080/10717544.2019.1704945

**Published:** 2019-12-20

**Authors:** Leen Van de Sande, Sarah Cosyns, Wouter Willaert, Wim Ceelen

**Affiliations:** a Laboratory of Experimental Surgery, Department of Human Structure and Repair, Ghent University, Ghent, Belgium;; b Cancer Research Institute Ghent (CRIG), Ghent University, Ghent, Belgium

**Keywords:** Albumin, intraperitoneal delivery, peritoneal metastasis, chemotherapy, peritoneum

## Abstract

Albumin is a remarkable carrier protein with multiple cellular receptor and ligand binding sites, which are able to bind and transport numerous endogenous and exogenous compounds. The development of albumin-bound drugs is gaining increased importance in the targeted delivery of cancer therapy. Intraperitoneal (IP) drug delivery represents an attractive strategy for the local treatment of peritoneal metastasis (PM). PM is characterized by the presence of widespread metastatic tumor nodules on the peritoneum, mostly originating from gastro-intestinal or gynaecological cancers. Albumin as a carrier for chemotherapy holds considerable promise for IP delivery in patients with PM. Data from recent (pre)clinical trials suggest that IP albumin-bound chemotherapy may result in superior efficacy in the treatment of PM compared to standard chemotherapy formulations. Here, we review the evidence on albumin-bound chemotherapy with a focus on IP administration and its efficacy in PM.

## 1. Introduction

The efficacy of a drug is dependent on the accumulation at the target site with a concentration and frequency that maximizes the therapeutic effectiveness and minimizes side-effects to the patient. Systemic chemotherapy is relatively inefficient in PM due to poor vascularity of the metastatic tumor nodules on the peritoneum (Tempfer, 2015; Winner et al., 2016). While IP drug delivery has been firmly established as a treatment option in patients with PM, clinical treatment has to rely on off-label use of drugs that were developed and approved for IV treatment. IP chemotherapy is based on the dose intensification provided by the delivery of chemotherapy into the peritoneal cavity and the delayed clearance caused by the peritoneal plasma barrier (Flessner et al., 1985; Dedrick & Flessner, 1997). Because of their activity profile, the taxanes are ideal candidates for IP administration. The potential of solvent-based paclitaxel (Sb-PTX, Taxol™) for IP administration is, however, limited by the local toxicity and potential of hypersensitivity reactions associated with the Cremophor EL™ solvent. Albumin-bound drug delivery has been utilized to overcome these obstacles. Nanoparticle albumin-bound paclitaxel (Nab-PTX, Abraxane^®^) was developed and demonstrated superior antitumor activity and less side-effects compared to Sb-PTX (Kinoshita et al., 2014). Likewise, methotrexate (MTX), 5-fluorouracil (5-FU), docetaxel and doxorubicin (DOX) were bound to albumin to improve the pharmacokinetics and pharmacodynamics of the drugs (Burger et al., 2001; D’Cruz et al., 2010; Maltas et al., 2016; Sharma et al., 2017). In theory, any cancer drug may be delivered IP but it is rational to explore IP delivery of albumin-bound chemotherapy. Cancers such as ovarian and pancreatic cancer express high levels of secreted protein acidic and rich in cysteine (SPARC), an albumin-binding 42-kDa matricellular glycoprotein whose expression in tumor interstitium correlates inversely with overall survival (Von Hoff et al., 2011). Albumin is a remarkable carrier with multiple cellular receptor and ligand binding sites, which promotes the delivery of chemotherapy in cancer cells. Here, we review albumin-bound cytostatics and their application in IP therapy.

## 2. The anatomy of the peritoneum

**Figure 1.**
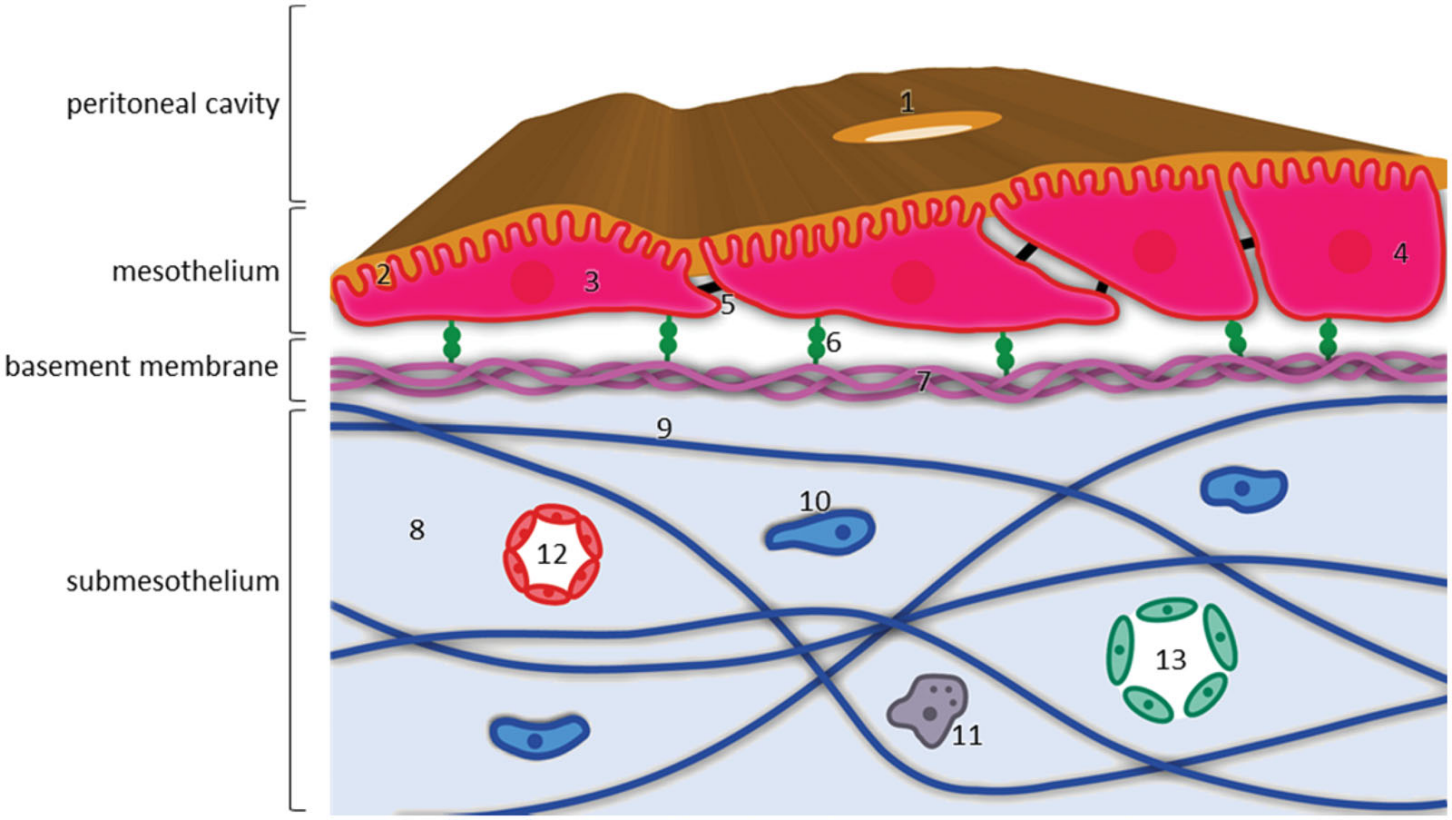
Structure of the peritoneum and underlying layers. The mesothelial monolayer covers the basement membrane and is supported by the submesothelial stroma. 1: stomata; 2: microvilli covered with a glycocalyx; 3: flattened mesothelial cell; 4: cuboidal mesothelial cell; 5: intercellular junction, mainly tight junction; 6: β1 integrin attached to basement membrane via laminin; 7: laminin and collagen IV fibers; 8: submesothelial stroma; 9: collagen, fibronectin, glycosaminoglycans and proteoglycans fibers; 10: fibroblast; 11: macrophage; 12: capillary; 13: lymphatic vessel.

The peritoneal cavity is contained within two leaves of a serosal membrane. The parietal peritoneum covers the abdominal wall, the pelvis, the anterior surfaces of the retroperitoneal organs and the inferior surface of the diaphragm, while the visceral peritoneum lines the intra-abdominal organs and mesenteries (Flessner, 2005). The total serosal exchange surface of the peritoneum is 1.5 m^2^ on average (Esquivel, 2010). Figure 1 illustrates the histology of the peritoneum. The peritoneum consists of a monolayer of flattened, squamous-like or cuboidal mesothelial cells supported by a basement membrane, a submesothelial connective tissue layer, and an underlaying cellular and associated microvessel network (Flessner, 2005; Mutsaers et al., 2016; Dakwar et al., 2017).

In normal conditions, the mesothelial cells are interconnected by tight junctions. On the apical surface of mesothelial cells, microvilli and cilia are present, which are covered in a glycocalyx consisting of proteoglycans and glycosaminoglycans. This glycocalyx secretes surface hyaluronan. As such, the mesothelial cells provide a non-adhesive surface and function as a barrier against physical damage (Flessner, 2005; Dakwar et al., 2017). On the subdiaphragmic peritoneal surface, lymphatic portals named stomata are abundantly present and interrupt the continuity of the mesothelial membrane. Stomata are located around the milky spots, maintaining a connection between the peritoneal cavity and the lymphatic system (van Baal et al., 2017). Mesothelial cells are anchored to the submesothelial basement membrane, the main components of which are collagen type IV and laminin. Mesothelial cells express β1 integrins to attach to the submesothelial basement membrane via laminin. The mesothelium is supported by submesothelial stroma through an extracellular matrix, consisting of collagen, fibronectin, glycosaminoglycans, and proteoglycans (van Baal et al., 2017).

PM occurs after a sequence of events, called the peritoneal metastatic cascade. After the metastatic tumor cells reach the peritoneal cavity, they are mobilized by the transport flow of peritoneal fluid. The adhesion of tumor cells might occur at several components of the peritoneum. The glycocalyx, mesothelial cell or the underlying stroma are targets for tumor cell adhesion. Tumor cells will breach the basement membrane after direct contact at places where the mesothelium is naturally discontinuous such as milky spots or disrupted by trauma, or due to mechanisms by which the tumor cell is able to denude the basement membrane. Further invasion of tumor cells is dependent on enzymatically degrading the extracellular matrix by matrix metalloproteinases (Sluiter et al., 2016). Peritoneal microvessels are hyperpermeable and pro-inflammatory cytokines and chemokines are secreted, leading to an oncotic pressure toward the peritoneal cavity. Tumor cells also induce apoptosis of the mesothelial cells leading to an altered structure of the peritoneal membrane (Flessner, 2005; Ceelen & Bracke, 2009; Sandoval et al., 2013).

**Figure 2.**
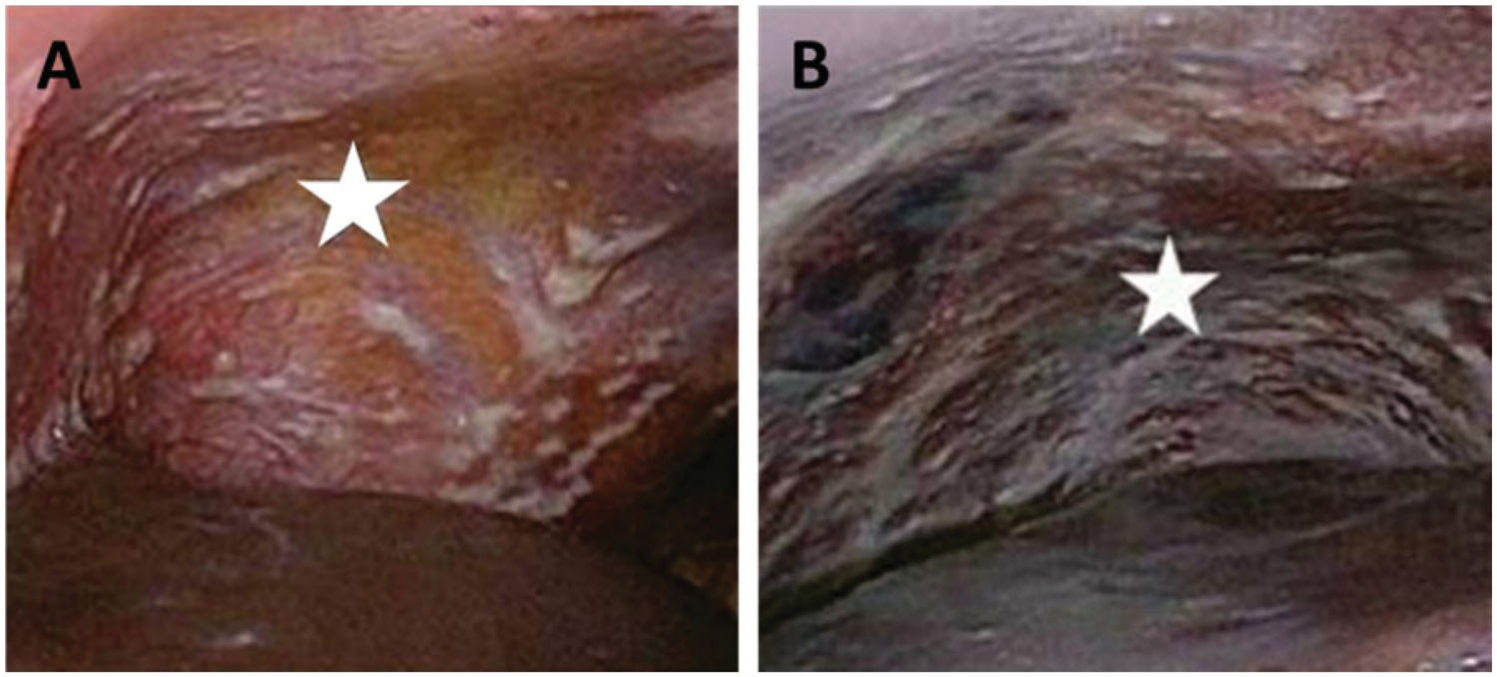
Irresectable peritoneal metastasis (white stars) in right upper abdomen (A) and left upper abdomen (B).

PM is a manifestation characterized by the presence of widespread metastatic tumor nodules on the peritoneum (Figure 2), originating from gastro-intestinal or gynaecological cancers (Coccolini, 2013). A small group of patients is eligible for surgical removal of all tumor nodules (debulking) combined with intraoperative chemoperfusion (Al Rawahi et al., 2013; Oseledchyk & Zivanovic, 2015). However, many patients present with irresectable disease, which has a dismal prognosis. Survival in patients with irresectable peritoneal metastases from colon cancer is 15 months, from gastric cancer 4 months and from pancreatic cancer only 6 weeks (Klaver et al., 2012; Thomassen et al., 2013; 2014). Systemic chemotherapy is relatively inefficient in PM due to poor vascularity of peritoneal tumor nodules (Tempfer, 2015; Winner et al., 2016).

## 3. Intraperitoneal drug delivery

### 3.1. The rationale for locoregional treatment

**Figure 3.**
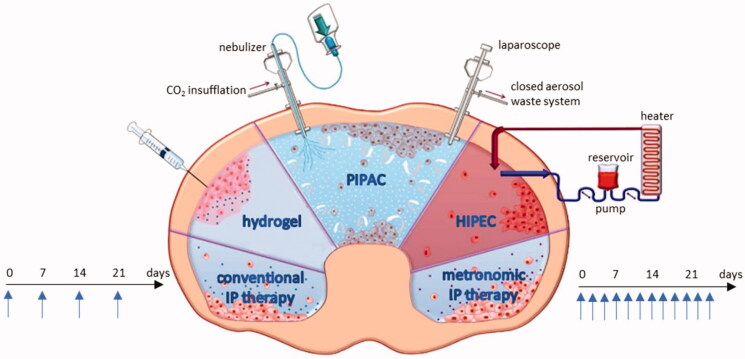
Overview of the strategies for IP drug delivery. IP: intraperitoneal; PIPAC: pressurized intraperitoneal aerosol chemotherapy; HIPEC: hyperthermic intraperitoneal chemotherapy. Figure adapted from Dakwar et al. (2017).

Locoregional therapy is based on the dose intensification provided by the administration of chemotherapy into the peritoneal cavity and the delayed clearance caused by the peritoneal plasma barrier (Flessner et al., 1985; Dedrick & Flessner, 1997). Figure 3 gives an overview of strategies for IP drug delivery. In addition to the conventional catheter-based strategy, metronomic dosing represents a novel approach defined as the frequent and continuous administration of conventional chemotherapy at low doses without drug-free breaks (André et al., 2014). Thermosensitive hydrogels containing drugs are another option for IP drug delivery. Hydrogels are liquid at room temperature but will form a gel at body temperature, leading to a prolonged exposure time (Fan et al., 2015). Another option involves intraoperative chemoperfusion (IPEC), immediately after cytoreductive surgery. Intraoperative chemoperfusion is usually performed under hyperthermic conditions (HIPEC) (Ceelen & Flessner, 2010). A recent method of IP drug delivery is pressurized intraperitoneal aerosol chemotherapy (PIPAC), which is performed during laparoscopy. The cytotoxic solution is injected under a maximal pressure of 20 bar, and the resulting aerosol is dispersed in the abdomen (Solass et al., 2014).

The peritoneal plasma barrier ensures a pharmacokinetic advantage leading to higher achievable IP concentrations whilst minimizing systemic toxicity (Dedrick et al., 1978; Dedrick & Flessner, 1997; Hasovits & Clarke, 2012). IP treatment also increases the drug concentration in the vicinity of avascular minimal peritoneal tumor nodules, which are difficult to eradicate with systemic chemotherapy (Dakwar et al., 2017). The pharmacokinetic advantage of IP drug delivery is usually expressed as the ratio of the area under the concentration-time curve (AUC) in the peritoneal over the plasma compartment (AUC_IP_/AUC_plasma_) and ranges widely from 2 to 1000 depending on the drug (Hasovits & Clarke, 2012; Carlier et al., 2017).

### 3.2. Ideal drugs for IP administration

The peritoneal barrier is a complex three-dimensional structure. Contrary to intuition, the mesothelial lining is not the main transport barrier, but the capillary walls and the surrounding interstitium are the most important barriers for the transport from the abdominal cavity to plasma (Flessner, 2005). Transport through the peritoneum was described by a mathematical formula where both plasma and the peritoneal cavity are considered as a single compartment separated from each other by an effective membrane:

$$\mathrm{rate}\;of\;\mathrm{mass}\;\mathrm{transfer}\;=PA\times (C_{p}\;-\;C_{B})$$

with PA the permeability area (effective contact area x permeability), C_p_ drug concentration in the abdominal cavity and C_B_ drug concentration in the blood (Dedrick & Flessner, 1997). The traditional two-compartment model of peritoneal transport describes transport of a drug from the peritoneal cavity to the blood crossing the peritoneal membrane, indicating that large molecular weight substances would be cleared more slowly from the peritoneal cavity than from the systemic circulation. This would increase drug exposure to the peritoneal tumor implants. The peritoneal clearance is inversely proportional to the square root of the molecular weight of the drug resulting in a higher concentration in the peritoneal cavity than in plasma after IP administration (Flessner et al., 1985).

Drugs that slowly exit the peritoneal cavity and that are rapidly metabolized during first passage through the liver, are more likely to exhibit a favorable pharmacokinetic advantage for cavity exposure after locoregional delivery, compared to drugs that do not exhibit these properties. Similarly, a biologically active drug, which is rapidly cleared from the systemic circulation after it enters the vascular compartment, will show a more favorable advantage than one that is slowly removed (either by metabolization into a nontoxic metabolite or elimination from the body by excretion through the kidneys). The drugs demonstrating the greatest difference between cavity and systemic exposures are drugs known to undergo extensive metabolism in the liver, e.g. 5-fluorouracil (5-FU), doxorubicin (DOX), cytarabine, paclitaxel (PTX), mitoxantrone (Kaplan et al., 1985; Goodman et al., 2016).

Unique toxicities must be considered with regional cytotoxic drug delivery. The peritoneal lining can be sensitive to the effects of cytotoxic drugs, leading to abdominal pain, sclerosis, and subsequent bowel obstruction (Kaplan et al., 1985; Walker et al., 2006; Graversen et al., 2018). Therefore, even if a drug is known to be active against the tumor type in question and preclinical models suggest promising pharmacokinetics and antitumor efficacy, clinical IP delivery may turn out to be precluded due to local toxic effects.

Table 1 provides an overview of the ideal drug characteristics for IP delivery. Recently, results from preclinical and early clinical trials have suggested that IP delivery of albumin-bound drugs may result in superior efficacy in the treatment of PM compared to the standard solvent-based formulation, whilst minimizing toxic side-effects (Kinoshita et al., 2014; Carlier et al., 2018; Cristea et al., 2019).

**Table 1.** Ideal drug characteristics for IP delivery (Helm & Edwards, 2007).

An ideal drug for IP delivery has the following characteristics:
inherent activity in the tumor type being treated; preclinical evidence for enhanced cytotoxicity associated with increasing either (or both) the peak concentration or total AUC versus time curve; not toxic to the peritoneal lining; extensively and rapidly metabolized to a nontoxic form during initial passage through the liver; quickly cleared after entry from the peritoneal cavity into the systemic compartment; drug does not require metabolism in the liver to become an active cytotoxic agent.

## 4. Albumin-based drug delivery

### 4.1. Properties of albumin

Albumin is the most abundant plasma protein in human blood with a concentration of 35–50 mg/mL and a molecular weight of 66.5 kDa. Albumin is synthesized in the liver hepatocytes with approximately 10–15 g of albumin produced and released in the vascular space daily (Larsen et al., 2016; Hoogenboezem & Duvall, 2018). When albumin extravasates into tissue, it is returned to the vascular space via the lymphatic system through a natural recycling mechanism. Interaction with cellular receptors is responsible for albumin’s cellular uptake and recycling. Albumin is known to be a carrier of a wide variety of both endogenous and exogenous compounds owing to its hydrophobic binding pockets (Kragh-Hansen, 1981). This facilitates the colloidal solubilization and transport of hydrophobic molecules such as fatty acids and steroids as well as different drugs. Furthermore, the surface of albumin is negatively charged making it highly water-soluble (Curry et al., 1998; Hoogenboezem & Duvall, 2018).

### 4.2. Albumin binding strategies

**Figure 4.**
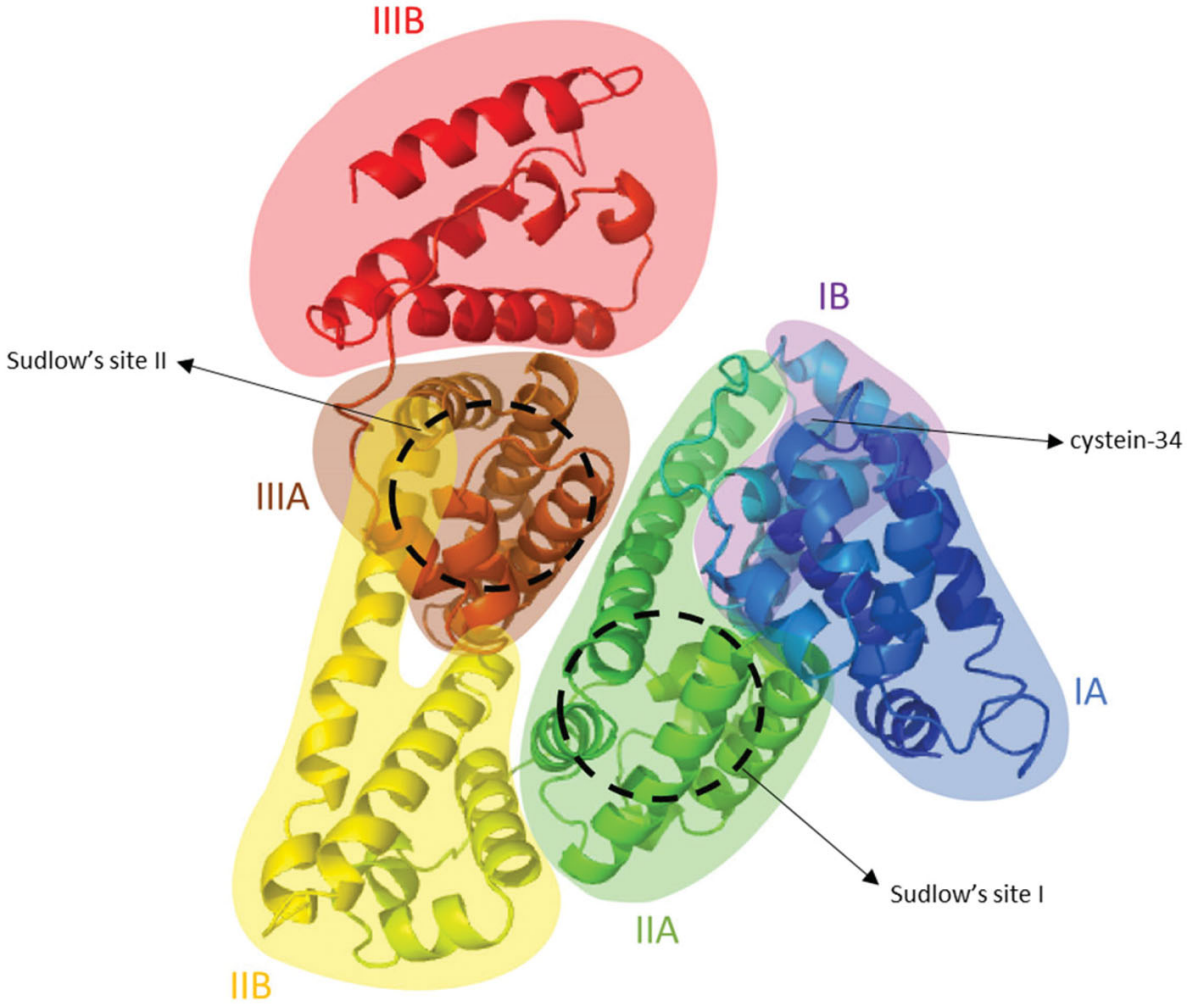
Cristal structure of HSA (PDB ID 1AO6). Albumin contains three homologous alpha helical domains I, II and III. Each domain is comprised of two subdomains A and B, which comprise four and six alpha-helices, respectively. Additional important binding sites include the free thiol located at the cysteine-34 amino acid residue and Sudlow’s sites I and II.

Structurally, albumin contains three homologous alpha helical domains I, II and III (Figure 4). Each domain is comprised of two subdomains A and B, which comprise four and six alpha-helices, respectively. Its seven fatty acid binding sites are distributed asymmetrically across the protein. Additional important binding sites include the free thiol located at the cysteine-34 amino acid residue and Sudlow’s sites I and II, which bind a variety of hydrophobic drugs. Of interest to the design of albumin-binding drugs is the distinct affinity and nature of each of these binding sites (Arroyo et al., 2014; Hoogenboezem & Duvall, 2018).

**Figure 5.**
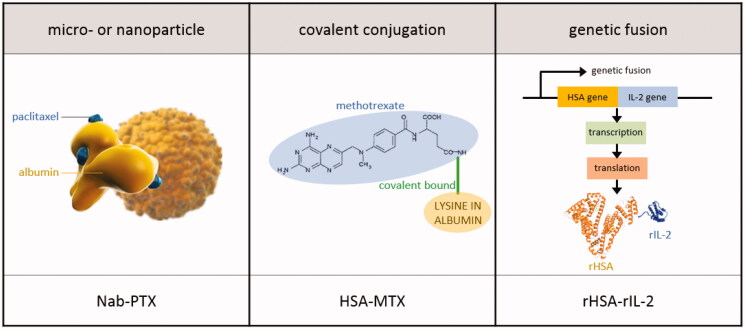
Albumin binding strategies. An example of an IP delivered drug per binding strategy is provided (Burger et al., 2001; Desai, 2016).

Hoogenboezem et al. defined two general binding strategies: preformed albumin therapeutics and in situ binders. Drugs that are categorized as in situ binders can dock on to circulating (endogenous) albumin after these drugs were delivered into the body. This facilitates transport, circulation time in blood and solubilization of hydrophobic drugs such as ibuprofen, diazepam, and warfarin (Kratz, 2008). In preformed formulations, (exogenous) albumin is attached to the drug prior to administration in the patient. Albumin was hereby isolated from human donors (human serum albumin; HSA), from bovine donors (bovine serum albumin; BSA) or recombinantly produced. As such, preformed formulations rely on drug loading into or attachment to exogenous albumin (Sjobring et al., 1988; Hoogenboezem & Duvall, 2018). This discussion will focus on exogenous albumin-based cancer therapeutics since in situ binders are not suitable for IP administration. Table 2 provides an overview of exogenous albumin-based cancer therapeutics. Based on the albumin binding strategy, exogenous albumin-based cancer therapeutics can be divided in micro- or nanoparticle formulations, covalent conjugations and genetic fusions (Figure 5).

**Table 2.** Overview of exogenous albumin-based cancer therapeutics.

Anti-tumoral compound	Drug name(s)	Binding strategy	Albumin	Clinical status	IP delivery	References
5-Fluorouracil	5-FU-rHSA-PEG-NP	Nanoparticle	rHSA	Preclinical in vitro	–	(Sharma et al., 2017)
17-Allylamino-17-demethoxygeldanamycin	ABI-010, Nab-17AAG	Nanoparticle	HSA	Withdrawn prior to phase I trial	–	(Desai, 2008; Larsen et al., 2016)
Curcumin	BSA-CCM	Covalent	BSA	Preclinical in vitro	–	(Sun et al., 2014)
Curcumin	HSA-CCM	Nanoparticle	HSA	Preclinical in vivo	–	(Kim et al., 2011; 2016)
Docetaxel	ABI-008, Nab-docetaxel	Nanoparticle	HSA	Phase I/II trial	–	(Desai, 2008; ClinicalTrials.gov, 2019b)
Docetaxel	ABI-013	Nanoparticle	HSA	Preclinical in vivo	–	(D’Cruz et al., 2010)
Doxorubicin	GA-rHSA-DOX	Nanoparticle	rHSA	Preclinical in vivo	–	(Qi et al., 2015)
Doxorubicin	L-HSA-DOX	Covalent	L-HSA	Preclinical in vivo	–	(Di Stefano et al., 2008)
Doxorubicin	Sp-HSA-DOX	Microparticle	Sp-HSA	–	–	(Maltas et al., 2016)
Lapatinib	–	Nanoparticle	HSA	Preclinical in vivo	–	(Wan et al., 2016)
Methotrexate	HSA-MTX	Covalent	HSA	Phase II trial	preclinical in vivo	(Hartung et al., 1999; Burger et al., 2001; Vis et al., 2002)
Paclitaxel	ABI-007, Nab-paclitaxel, Abraxane^®^	Nanoparticle	HSA	FDA and EMA approved (IV)	phase I/II trial	(Desai, 2008; Gardner et al., 2008; Xiao et al., 2009; Von Hoff et al., 2011; Coccolini, 2013; Kinoshita et al., 2014; Kim et al., 2016; Carlier et al., 2018; Van De Sande et al., 2018; Cristea et al., 2019)
Proaerolysin	–	Genetic fusion	HSA	Preclinical in vivo	–	(Pruitt et al., 2016)
Rapamycin	ABI-009, Nab-rapamycin	Nanoparticle	HSA	Phase II trial	–	(Desai, 2008; ClinicalTrials.gov, 2019c)
Recombinant interleukin-2	Albuleukin, rHSA-rIL-2	Genetic fusion	rHSA	Phase I trial	preclinical in vivo	(Osborn et al., 2004; Melder et al., 2005)
Thiocolchicine dimer	ABI-011, Nab-5404	Nanoparticle	HSA	Phase I trial	–	(Desai, 2008; ClinicalTrials.gov, 2019a)

### 4.3. Albumin-bound anticancer therapeutics

#### 4.3.1. Micro- or nanoparticle formulations

A method that utilizes albumin as a carrier for cancer therapeutics involves drug encapsulation into an exogenous albumin-based particle. The methods for synthesizing albumin particles can be generally categorized into the techniques of desolvation, emulsification, thermal gelation, nano-spray drying and self-assembly (An et al., 2014; An & Zhang, 2017; Hoogenboezem & Duvall, 2018).

Nab-technology is a patented novel nanotechnology-based drug delivery platform developed by Abraxis BioScience (currently under tradename Celgene, New Jersey, United States), which exploits the natural properties of albumin to achieve a safe, solvent-free, efficient and targeted drug delivery (Desai, 2008). Nab-PTX is approved by the United States Food and Drug Administration (FDA) and European Medicines Agency (EMA) for intravenous (IV) treatment of metastatic breast cancer, locally advanced or metastatic non-small cell lung cancer, and metastatic pancreatic cancer in combination with gemcitabine (Desai, 2008; Gardner et al., 2008; Von Hoff et al., 2011). Nab-rapamycin was developed to treat non-muscle invasive bladder cancer and Nab-docetaxel proved to be effective against prostate and metastatic breast tumors (Desai, 2008; ClinicalTrials.gov, 2019b,c). Nab-CY196 is a novel albumin nanoparticle (NP) docetaxel analog with an improved activity and safety profile compared to Nab-docetaxel (D’Cruz et al., 2010). Another albumin-based NP contains the Hsp90 inhibitor 17-allylamino-17-demethoxygeldanamycin (17-AAG). A phase I trial (NCT00820768) was planned with this therapeutic in combination with Nab-PTX for advanced non-hematologic malignancies, but the study was withdrawn prior to enrollment for an unknown reason (Desai, 2008; Larsen et al., 2016). Nab-5404 comprises a novel thiocolchicine dimer that possesses dual inhibition of tubulin polymerization and topoisomerase I activities and exhibits antiangiogenic and vascular targeting activities leading to cytotoxic efficacy against solid tumors and lymphomas (Desai, 2008; ClinicalTrials.gov, 2019a). This exhaustive list of drugs based on Nab-technology is currently under research.

In addition to the drugs formulated by Abraxis Bioscience, many other labs have experimented with the delivery of hydrophobic small-molecule anticancer drugs using albumin particles. In 2011, Kim et al. (2011) fabricated a curcumin (CCM)-loaded HSA NP using Nab-technology. Curcumin is a pharmacologically active polyphenolic compound present in *Curcuma longa* (turmeric) and is traditionally used as a natural spice. CCM inhibits nuclear factor-kappa beta (NF-κβ), which is involved in the pathogenesis of several malignancies and inhibits production of cytokines such as tumor necrosis factor α (TNF-α) and interleukin-1β (IL-1β). In vitro and in vivo studies have shown cytotoxicity against colon and pancreatic tumor cells. In 2016, a co-loading of PTX and CCM via Nab-technology using high-pressure homogenization was described. The PTX/CCM albumin NPs demonstrated in vitro anti-tumor efficacy against pancreatic cancer cells (Mia Paca-2 cells) (Kim et al., 2016). Co-encapsulation of CCM and doxorubicin (DOX) in albumin NPs was tested on MCF-7 resistant breast cancer cells. DOX/CCM albumin NPs blocked the adaptive treatment tolerance of cancer cells and elicited efficient cell killing (Motevalli et al., 2019). Similarly, lapatinib-loaded HSA NPs were described by Wan et al. (2016). Lapatinib is a selective small-molecule dual-tyrosine kinase inhibitor (TKI) of the human epidermal growth factor receptor 2 (HER2) and the epidermal growth factor receptor (EGRF). Lapatinib loaded HSA NPs showed in vivo efficacy against triple negative breast cancer and also prevented breast cancer metastasis to the brain. 5-FU was conjugated to polyethylene glycol (PEG) anchored recombinant HSA (rHSA) NPs (5-FU-rHSA-PEG-NPs). Preclinical in vitro experiments suggested improved cytotoxicity and pharmacokinetic profiles compared to 5-FU using a human colon cancer cell line (HT-29) (Sharma et al., 2017).

Albumin NPs can be decorated with a variety of targeting ligands to give additional specificity to cancer cell-associated receptors. For instance, anti-cancer drugs were loaded into mannosylated bovine serum albumin (BSA) NPs to target drug-resistant colon cancer cells and tumor-associated macrophages, which both highly express mannose receptors and SPARC (Zhao et al., 2017). Likewise, folate-decorated BSA NPs were developed for the targeted delivery of PTX to exploit overexpression of the folate receptor by a wide range of tumor cell types (Zhao et al., 2010). The glycyrrhetinic acid (GA) receptor is overexpressed in liver cancer cells. Consequently, GA modified rHSA NPs were developed to target liver tumor cells. Qi et al. encapsulated GA-rHSA NPs with DOX (GA-rHSA-DOX) and demonstrated increased cytotoxic activity in liver tumor cells compared to non-targeted NPs (rHSA-DOX) (Qi et al., 2015). Albumin NPs can also be decorated with antibodies such as DI17E6, a monoclonal antibody directed against αv integrins, which are cell membrane-spanning matrix adhesion domains that are highly expressed in various cancer lines. Covalent coupling of DI17E6 onto DOX loaded albumin NPs showed inhibited growth and angiogenesis in melanoma (Wagner et al., 2010). Yu et al. (2016) described albumin NPs decorated with cyclic arginine-glycine-aspartic (cRGD) peptides loaded with gemcitabine for the treatment of pancreatic cancer. The α_v_β3 integrins specifically recognize the cRGD motif which suggests the possibility of using cRGD-conjugated carriers to deliver drugs into cancer cells as active tumor targeting therapy. Finally, a sporopollenin-HSA (Sp-HSA) microparticle was developed as a drug carrier. The Sp-HSA particles were loaded successfully with DOX for targeted cancer treatment (Maltas et al., 2016). To date, anti-cancer efficacy studies for these Sp-HSA particles are lacking.

#### 4.3.2. Covalent conjugations

Common strategies for direct, covalent conjugation involve binding of the drug to either lysines, tyrosines or the free SH-group on the cysteine-34 amino acid residue of albumin (Larsen et al., 2016; Hoogenboezem & Duvall, 2018). HSA methotrexate (HSA-MTX) is a covalent-bound MTX to lysine residues in albumin. This conjugate was developed to improve the pharmacokinetic profile of MTX. Methotrexate conjugated at a 1:1 HSA:MTX ratio showed significant anti-cancer efficacy in sarcoma as well as in prostate xenograft models (Burger et al., 2001). A phase II clinical trial showed that HSA-MTX in combination with cisplatin was effective against urothelial carcinomas with an acceptable toxicity profile (Hartung et al., 1999). However, no objective responses were seen in patients with metastatic renal cell carcinoma who had progressed after previous immunotherapy (Vis et al., 2002). Similar to MTX, DOX was covalently conjugated with lactosaminated human albumin (L-HSA) to increase its efficacy in the treatment of hepatocellular carcinoma. The anti-cancer efficacy of L-HSA-DOX was compared to unbound DOX in a preclinical experiment. Compared to control rats treated with saline, L-HSA-DOX significantly reduced the number of neoplastic nodules, whereas the free DOX administered at the same dose was ineffective. Moreover, free DOX markedly decreased the body weight of rats, a sign of systemic toxicity, which was not caused by L-HSA-DOX (Di Stefano et al., 2008). In 2014, Sun et al. (2014) reported a BSA-CCM conjugate. The anti-cancer activity of free CCM and BSA-CCM conjugate was assessed by an MTT assay on HeLa cells. Only BSA-CCM conjugate showed significant inhibitory effect against HeLa cells. Free CCM and its derivatives were insoluble in water and could therefore not inhibit the growth of HeLa cells. In contrast, BSA-CCM was readily soluble in water amplifying the bioactivity against HeLa cells and inhibiting cellular proliferation.

#### 4.3.3. Genetic fusions

Albumin fusion proteins are created by fuzing the gene that expresses albumin to the gene that expresses a therapeutically active protein (Dou et al., 2008). Pruitt et al. (2016) produced a rHSA linked to the N-terminus of proaerolysin via a peptide linker specific for the protease prostate specific antigen. This pro-toxin can only be cleaved, and thus activated, by a defined protease that is present in the prostate tumor micro-environment. Recombinant interleukin-2 (rIL-2) is thought to mediate anti-tumor cellular immune responses through lymphocyte activation and is currently a therapy for melanoma and renal cell carcinoma (Rosenberg, 2001). A rIL-2 was genetically fused to rHSA creating the albuleukin fusion protein. Albuleukin was introduced in clinical practice to assess its therapeutic benefit in a variety of cancers (Melder et al., 2005).

### 4.4. Transport mechanism of albumin-based drugs

Albumin is an important carrier protein with a number of putative albumin-binding proteins and receptors that have been identified in various tissues and cell lines. Table 3 provides a summary of albumin-binding proteins and receptors. Unfortunately, there are relatively few papers studying the cellular receptors of albumin and the significance of these results were found to be mostly unclear (Merlot et al., 2014). Consequently, further research is necessary to validate the locations and functions of albumin-binding proteins and receptors. This review will further focus on the transport mechanisms of albumin-based drugs after IV and IP administration.

**Table 3.** Overview of cellular receptors and ligand binding sites of albumin (Merlot et al., 2014; Chatterjee et al., 2017; Infante et al., 2007).

Albumin-binding proteins	Tissue	Function
cubilin	Kidney, intestines, placenta, york-sac cells	Endocytosis and transcellular transport of albumin; reabsorption of albumin in kidney proximal tubule cells
FcRn	Endothelium, antigen-presenting cells, intestines, kidney, lung, blood-brain-barrier	Protection of albumin from degradation in acidic endosomes and returns albumin to the extracellular space
gp18	Endothelium, macrophages, fibroblasts, tumor	Bind and direct modified albumin for degradation
gp30	Endothelium, macrophages, fibroblasts, tumor	Bind and direct modified albumin for degradation
gp60	Endothelium	Internalization and transcytosis of albumin
hnRNP family	Tumor	Involved in pre-mRNA processing; cell adhesion, modulation of platelet collagen interactions, apoptosis (calreticulin)
megalin	Kidney, intestines, placenta, york-sac cells, choroid plexus, thyrocytes, epithelium, lung, parathyroid, endometrium, oviduct, inner ear, epididymal cells	Contributes to the internalization of cubilin-ligand complexes as a co-receptor; reabsorption of albumin in kidney proximal tubule cells
SPARC	Endothelial cells, vascular smooth muscle cells, skeletal muscle, fibroblasts, testicle, ovary, pancreas, tumor	Accumulation of albumin-bound drugs within tumor interstitium

#### 4.4.1. IV Administration

**Figure 6.**
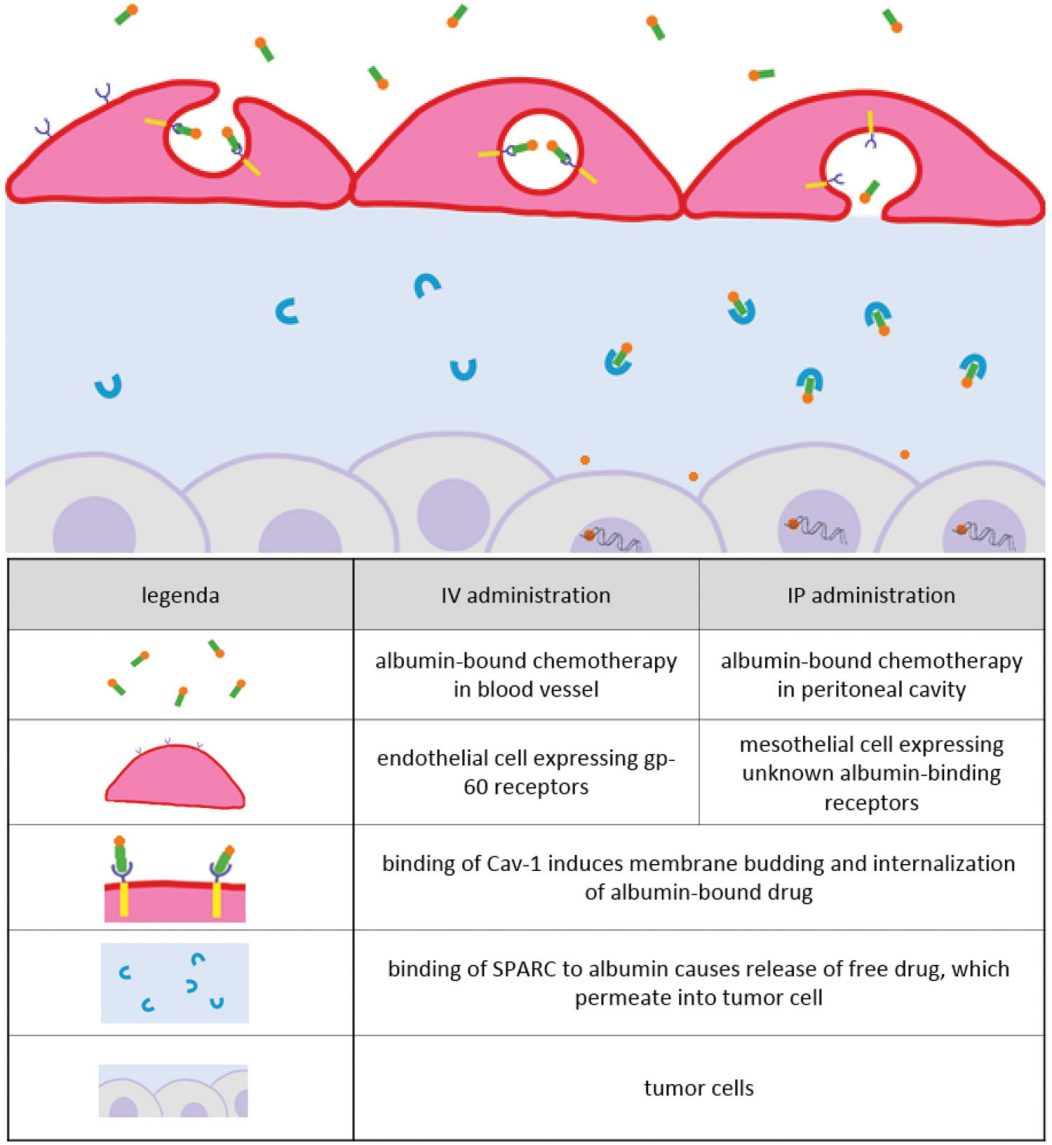
Receptor-mediated transcytosis of albumin-based drugs after IV or IP administration.

Transport of albumin-based drugs after IV administration is well described (Figure 6). Transcytosis of albumin across the endothelium of blood vessels is mediated by gp60, a 60-kDa glycoprotein localized on the endothelial cell surface that binds albumin with high affinity in the nanomolar range. The binding of albumin to gp60 induces gp60 clustering and association with caveolin-1 (Cav-1), leading to the formation of caveolae that will carry the albumin complexes from the apical to the basal membrane, where the caveolae content is released into the tumor interstitium. Binding of SPARC to albumin causes release of free drug, which permeates into tumor cells.

Receptor-mediated albumin uptake by cancer cells has become evident based on the correlation between the expression of albumin-related receptors and the efficacy of albumin-bound drugs among different cancer types. Chatterjee et al. attempted to demonstrate why certain patients responded better to a treatment with Nab-PTX than others. In preclinical in vivo experiments, Cav-1 protein levels correlated positively with Nab-PTX sensitivity. RNAi-mediated attenuation of Cav-1 expression reduced uptake of albumin and Nab-PTX in cancer cells and rendered them resistant to Nab-PTX-induced apoptosis. Conversely, Cav-1 overexpression enhanced sensitivity to Nab-PTX (Chatterjee et al., 2017). Zhao et al. (2018) further specified that higher tumor Cav-1 levels and lower stromal Cav-1 levels were significantly associated with longer progression free survival of metastatic breast cancer patients receiving Nab-PTX in combination with gemcitabine. It has been hypothesized that the accumulation of albumin in the tumor interstitium is facilitated by SPARC. This hypothesis was based on a clinical trial with gemcitabine and Nab-PTX in patients with advanced pancreatic cancer. SPARC levels were evaluated in thirty-six patients. An increase in SPARC levels was correlated with improved overall survival. The significant increase in SPARC levels was limited to the stroma and was not present in tumor cells (Von Hoff et al., 2011). This finding suggested that the presence of SPARC in the tumor interstitium would concentrate Nab-PTX and thus enhance its therapeutic effect. However, other preclinical and clinical experiments showed no correlation between SPARC levels and treatment efficacy (Guweidhi et al., 2005; Infante et al., 2007; Hidalgo et al., 2015; Chatterjee et al., 2017). Other albumin-binding proteins and receptors that may mediate the accumulation of albumin-bound carriers in the tumor (interstitium) include gp18, gp30, calreticulin, megalin, cubilin, heterogeneous nuclear ribonucleoproteins (hnRNPs), and the neonatal Fc receptor (FcRn) (Merlot et al., 2014).

#### 4.4.2. IP administration

The peritoneal membrane is frequently considered to form a barrier to albumin resorption. However, mechanistic analyses have proven this assumption to be incorrect. Studies in rodents and in dialysis patients have shown that proteins leave the peritoneal cavity at rates 5–10 times the rate in which it appears in plasma (Daugirdas et al., 1980; Flessner et al., 1983; Kumano et al., 1996). Through dissection of rodent tissues, it has been demonstrated that all the proteins that left the peritoneal cavity but did not reach the plasma were contained in the surrounding peritoneal tissues (Flessner et al., 1983; Flessner & Schwab, 1996). In addition, subsequent experiments showed that the rate of protein transfer was quantitatively the same as the rate of fluid transfer of an isotonic solution administered in the peritoneal cavity (Flessner & Schwab, 1996). Moreover, the extent of parietal peritoneal resection did not affect the pharmacokinetics of intraoperative IP chemotherapy illustrating that the pharmacological barrier between the peritoneal cavity and plasma is not directly related to an intact peritoneum (de Lima Vazquez et al., 2003). Consequently, Flessner (2005) concluded that the peritoneum is a very loose barrier.

The mechanisms and pathways governing the peritoneal absorption of albumin from the peritoneal cavity have not been completely identified (Figure 6). Gotloib & Shostak (1995) injected healthy mice IP with BSA-gold particles to assess transmesothelial absorption. A significant higher proportion of the BSA-gold particles was detected in transcytotic vesicles versus in intermesothelial junctions, supporting the idea of a continuously transcytotic mechanism transporting albumin across the mesothelial layer. In this sense, transcytotic vesicles could represent the large pore equivalent, similar to transport after IV administration. These findings are in accordance with the experiments of Bodega et al. (2002) who investigated the transport of albumin through the mesothelium of parietal pericardium. Fresh retrosternal parietal pericardium of rabbits was isolated and mounted as planar sheets in an Ussing chamber containing I^125^-albumin solution. Thereafter, I^125^-albumin was detected in the mesothelial cells of parietal pericardium by scintillation spectrometry. The results showed the occurrence of an active transport of albumin from the luminal to the interstitial side of the mesothelium. This active transport was due to transcytosis. Moreover, the results demonstrated that transcytosis decreases progressively at low albumin concentrations (C_alb_) and eventually vanishes when C_alb_≤0.005%. Transcytosis ceased when an inhibitor for transcytosis (40 µM nocodazole) was added to a 0.5% C_alb_ solution. This suggests that the vesicular transport is not constitutive but appears to be activated by albumin. Therefore, transmesothelial transport of albumin-bound complexes probably occurs via transcytosis, similar to transendothelial transport. The question arises whether the mechanisms of albumin transcytosis in mesothelium and endothelium utilize the same albumin-binding proteins and signaling pathways. To our knowledge, nothing is known about the mechanisms of cellular receptor-mediated albumin transport in mesothelial cells.

The findings of Gotloib and Bodega were based on experiments performed on healthy mesothelium, in which the role of the intermesothelial cell junctions seemed to be minor. This transport pathway may, however, become highly permeable to anionic plasma proteins in a tumor tissue environment. The tight junctions and basement membrane are disrupted by exposure of the mesothelium to inflammatory mediators such as hepatocyte growth factor (HGF). Tumor cells induce apoptosis of the mesothelial cells leading to an altered structure of the peritoneal membrane (Flessner, 2005; Ceelen & Bracke, 2009). This results in the peritoneum becoming a looser barrier to albumin. Therefore, to unravel the transport mechanisms of albumin-bound drugs after IP administration, further research should also focus on intracellular transport of albumin-bound drug in this specific environment.

## 5. Efficacy of albumin-based drugs after IP administration

Evidence for the efficacy of albumin-based drugs after IP administration has been demonstrated for Nab-PTX, HSA-MTX, rHSA-rIL-2 and HSA-Au NPs. Table 4 gives an overview of the comparative preclinical experiments.

**Table 4.** Efficacy of albumin-based drugs after IP administration.

Drugs	Experimental setup	Evidence after IP delivery	References
Nab-PTX	Mouse gastric cancer xenograft	Survival was higher in the Nab-PTX treatment group (126 days) compared to the Sb-PTX treatment group (96 days).	(Kinoshita et al., 2014)
	Mouse ovarian cancer xenograft	Survival was higher in the Nab-PTX treatment group (81 days) compared to the Sb-PTX treatment group (65 days).	(Xiao et al., 2009)
	Mouse ovarian cancer xenograft	Nab-PTX led to more pronounced tumor penetration and tumor cell death compared to mic-PTX.	(Carlier et al., 2018)
	Non-tumor bearing rabbits	PTX after Nab-PTX treatment penetrated up to 0.63 mm in the peritoneal wall, but after Sb-PTX, PTX was not detectable in the peritoneum. The peritoneal concentration after IP Nab-PTX delivery was five times higher compared to Sb-PTX.	(Desai, 2016)
HSA-MTX	Mouse soft tissue sarcoma xenograft	A single IP injection of MTX-HSA caused complete tumor remission for more than 119 days. Repeated IV injections of MTX resulted in short-lasting partial tumor regression.	(Burger et al., 2001)
	Mouse prostate cancer xenograft	MTX-HSA showed tumor growth inhibition of 92.8% compared to the control mice, while injection of MTX showed growth inhibition of 20.8% compared to the control mice.	(Burger et al., 2001)
rHSA-rIL-2	Mouse renal cancer allograft	Tumor volume was decreased to 280 mm³ in the rHSA-rIL-2 treatment group, compared to 1320 mm³ in the rIL-2 treatment group. The survival of the treatment groups was similar.	(Melder et al., 2005)
HSA-Au NPs	Mouse colon cancer allograft	Accumulation of Au-HSA NPs in the peritoneal cavity and tumor lesion after IP injection was higher, compared to IV injection. After IP injection, AUC of ascites and tumor were respectively 93- and 20-fold higher, while the AUC of liver and spleen were respectively 12- and 11-fold lower, compared to IV injection.	(Chen et al., 2019)

### 5.1. Nab-PTX

The taxanes are ideal candidates for IP administration due to their activity profile and molecular size. Standard formulation of PTX is highly hydrophobic and thus requires the use of solvents such as Cremophor-EL, which contribute to some of the toxicities commonly associated with PTX-based therapy (Stinchcombe, 2007). Nab-PTX is a solvent-free formulation of PTX. Binding PTX to albumin by high-pressure homogenization of PTX in the presence of serum albumin into a NP colloidal suspension has several practical advantages over Sb-PTX, such as the diminished need for premedication to prevent hypersensitivity reactions. The Nab-PTX formulation eliminates the impact of Cremophor-EL on PTX pharmacokinetics and utilizes the endogenous albumin transport mechanisms to concentrate Nab-PTX in the tumor, leading to improved anti-cancer efficacy (Stinchcombe, 2007; Kinoshita et al., 2014; Carlier et al., 2018).

Kinoshita et al. (2014) reported a comparative in vivo study to evaluate the antitumor activity of Nab-PTX and Sb-PTX after IP administration. Female athymic NCr-nu nude mice were simultaneously inoculated with 1 × 10^7^ OCUM-2MD3 cells, a high peritoneal-seeding cell line from human gastric cancer. The tumor-bearing mice were divided into three groups: a control group, a Nab-PTX treatment group, and a Sb-PTX treatment group. Antitumor activity was compared among these three groups. After tumor inoculation on day 0, drug treatment was initiated on day 7, and drug was administered once daily for seven consecutive days at equitoxic doses. Nab-PTX treatment resulted in a significantly higher antitumor activity compared to Sb-PTX treatment. All five mice in the control group developed ascites and died within 19–32 days after tumor cell inoculation, with a median survival of 25 days. Animal survival was significantly better in the Nab-PTX treatment group (median 126 days) compared to that of the Sb-PTX treatment group (median 96 days).

These findings were in line with the results obtained by Xiao et al. (2009). An orthotopic intraperitoneal model of metastatic ovarian cancer was developed by injecting 1 × 10^7^ luciferase positive SK-OV-3 cells IP into nude mice. Sb-PTX, Nab-PTX or PBS (control) was intraperitoneally injected on day 0, 4, 8, 12, and 16 at equitoxic doses. Bioluminescence imaging was performed weekly after the treatment. Strikingly, none of the mice treated with Sb-PTX demonstrated complete response. Median overall survival was 39 days for untreated mice in the PBS control group, while the Sb-PTX treated mice showed a median overall survival of 65 days. Therapy with Nab-PTX further prolonged the overall survival to 81 days.

Similarly, the preclinical activity of Nab-PTX and polymeric micellar PTX (mic-PTX) were tested in athymic nude Foxn1^nu^ mice (Carlier et al., 2018). All mice were bilaterally engrafted in the subperitoneal space with 5 × 10^5^ luciferase positive SK-OV-3 cells to create peritoneal ovarian cancer xenografts. Drug treatment was initiated 2 weeks after tumor cell inoculation. The xenografts were then treated with repeated IP injections of Nab-PTX, mic-PTX or saline (control). Both PTX formulations significantly reduced the number and volume of peritoneal tumor nodules and prolonged survival, compared to the control group. The mitotic index was significantly increased after IP Nab-PTX, but no difference was observed between the IP mic-PTX group and the control group. Four hours after IP injection, matrix-assisted laser desorption/ionization (MALDI) imaging showed homogeneous and extensive tumor tissue penetration of Nab-PTX, which was not observed in the mic-PTX treatment group. Compared to mic-PTX, Nab-PTX lead to more pronounced tumor penetration and tumor cell death. This may be explained by the slow release in the peritoneal cavity of PTX from the micellar formulation, since hydrophobic PTX tend to remain in the hydrophobic core of polymeric micelles. In contrast, Nab-PTX will more easily dissociate after IP administration due to the reversible non-covalent binding (Miele et al., 2009).

In a recent study using a hyperthermic IP chemotherapy (HIPEC) model in the rabbit, Nab-PTX was compared to Sb-PTX (Coccolini et al., 2017). Samples of perfusate and blood were collected at different time points and peritoneal tissues were collected at the end of perfusion. PTX after Nab-PTX treatment penetrated up to 0.63 mm in the peritoneal wall, but after Sb-PTX, PTX was not detectable in the peritoneum. Moreover, the peritoneal concentration after IP Nab-PTX delivery was five times higher compared to Sb-PTX. Despite the high levels reached in the peritoneum, systemic exposure of PTX remained low.

IP catheter-based delivery of Nab-PTX was recently studied in a phase I clinical trial in advanced carcinomatosis patients to determine the maximal tolerated dose (MTD) (Cristea et al., 2019). Nab-PTX was administered weekly on days 1, 8, and 15 of a 28-day cycle in successive cohorts of patients with no intra-patient dose escalation. Doses explored were 35, 70, 90, 112.5, 140, and 175 mg/m^2^. No dose-limiting toxicities (DLTs) were observed in dose levels 35, 70, and 90 mg/m^2^. A DLT was noticed in one of six patients in dose level 112.5 mg/m^2^ (grade 3 neutropenia causing more than 15 days treatment delay) and a DLT in one of three patients allocated to dose level 175 mg/m^2^ (grade 4 neutropenia and grade 3 abdominal pain). A second patient in dose level 175 mg/m^2^ experienced a serious adverse event (cycle 1 grade 4 neutropenia less than 7 days, cycle 4 grade 2 left ventricular dysfunction). This dose level was determined to be above the MTD. No DLTs were seen in all patients treated with 140 mg/m^2^ Nab-PTX. Therefore, the MTD of IP Nab-PTX was established at 140 mg/m^2^. A significant pharmacokinetic advantage of IP Nab-PTX was found at each dose level. Across all dose levels of Nab-PTX, the median IP versus IV AUC was 147-fold, resulting in increased peritoneal drug exposure. Eight of twenty-seven enrolled patients showed a progression free survival of more than 6 months. One patient experienced a complete response, and one patient experienced a partial response. Six patients had stable disease.

Recently, the technique of laparoscopic (pressurized) IP aerosol chemotherapy (PIPAC) was introduced in clinical practice (Solass et al., 2014; Grass et al., 2017). During laparoscopy, chemotherapy is delivered as an aerosol, generated by a dedicated micropump connected to a high-pressure injector. Advantages of PIPAC include minimal patient discomfort, the possibility of repeated delivery, the potential to combine it with systemic treatment, and the possibility to assess pathological response of peritoneal disease by serial biopsies. In theory, any cancer drug may be delivered IP as an aerosol. A multicenter, first-in-human phase 1 dose escalation study to explore the safety of PIPAC using Nab-PTX in patients with unresectable peritoneal metastasis was initiated (NCT03304210) (Van De Sande et al., 2018). Patients will undergo three consecutive PIPAC procedures with an interval of 4 weeks. The dose levels of Nab-PTX are 35, 70, 90, 112.5, and 140 mg/m^2^. The same dose will be used for all three treatments in the same patients.

### 5.2. HSA-MTX

After IV administration, MTX is rapidly and efficiently cleared from the circulation. The mean distribution half-life ranges from 1.5 to 3.5 h in patients with normal total body clearance (Evans et al., 1986). Consequently, tumor exposure time of MTX is short, and a HSA-MTX conjugate was introduced to prolong exposure. A comparative in vivo study examined the antitumor activity of HSA-MTX (12.5 mg/kg) after IP administration versus IV administration of unbound MTX (100 mg/kg) (Burger et al., 2001). A soft tissue sarcoma xenograft (SXF 1301) and a prostate-cancer xenograft (PRXF PC3M) were used. Tumor fragments of 25 mg were subcutaneously (SC) implanted in both flanks of outbred nude mice. When tumors were clearly palpable and had reached a volume of 100–200 mm^3^, mice were randomly allocated into treatment groups and were weekly treated for 3 weeks. In the soft tissue sarcoma xenograft, a single IP injection of MTX-HSA was sufficient to cause complete tumor remission for more than 119 days (end of experiment) after treatment was initiated. Therefore, injections on days 8 and 15 were not given. IV MTX was less effective and resulted in only short-lasting partial tumor regression. In the prostate-cancer xenograft, MTX-HSA showed tumor growth inhibition of 92.8% compared to the control mice, while injection of MTX showed growth inhibition of 20.8% compared to the control mice.

### 5.3. rHSA-rIL-2

Interleukin-2 is thought to mediate antitumor cellular immune responses through lymphocyte activation, and is currently approved for the IV treatment of melanoma and renal cell carcinoma (Rosenberg, 2001). However, the short half-life of rIL-2 and its systemic toxicity continue to limit the clinical use of this recombinant protein (Lotze et al., 1985). Albumin fusion technology provides the advantageous pharmacokinetic properties of albumin to a fusion partner such as rIL-2, resulting in a new protein with improved therapeutic potential. The pharmacological activity of rHSA-rIL-2 was examined in female BALB/c mice to determine whether the fusion protein had the immunomodulatory and antitumor properties of rIL-2 (Melder et al., 2005). On day 0, mice were inoculated SC in the midflank region with 1 × 10^5^ Renca cells, a murine renal carcinoma cell line. Mice received daily IP injections of rIL-2 (0.9 mg/kg) on days 10–14 and 17–21. Control mice received daily IP injections with PBS on the same days. The effect of rHSA-rIL-2 (0.6 mg/kg) was evaluated by IP injection on days 12, 14, 16, 19, 21, and 23. Tumor volume was measured on day 28 using millimetre-calibrated calipers and mice were monitored for survival on a daily basis until 40 days post-inoculation. On day 28, median tumor volume was 3200 mm^3^ in the control group, while the rIL-2 treated mice showed a non-significant decrease to a median volume of 1320 mm^3^. The median tumor volume further decreased to 280 mm^3^ in rHSA-rIL-2 treated mice, which was significantly smaller compared to the control group. In addition, three of ten mice treated with rHSA-rIL-2 were either tumor-free or had minimally detectable tumor (<1 mm^3^) compared to zero of ten mice in the control group. Four out of ten control mice survived until day 28 while all mice receiving rHSA-rIL-2 survived. The survival benefit after IP treatment of rIL-2 was similar to that of rHSA-rIL-2.

### 5.4. HSA-Au NPs

Hybrid protein-inorganic NP systems have displayed multifunctional applications in solid cancer theranostics (An et al., 2014; An & Zhang, 2017). However, the potential of these NPs for treating peritoneal metastases remains unclear. Chen et al. developed a gold nanocore-encapsulated HSA (Au-HSA) NP as a drug delivery system (Chen et al., 2019). Its radioactive surrogate Indium-111 labeled Au-HSA (^111^In-Au-HSA) was prepared to investigate the biological behavior in a CT-26 colon tumor/ascites-bearing mouse model. Male BALB/c mice were inoculated intraperitoneally with 2 × 10^5^ CT-26 cells, a murine colon carcinoma cell line. Ten to 14 days after tumor cell inoculation, mice received ^111^In-Au-HSA NPs by either an IV or IP injection. Both biodistribution and microSPECT imaging exhibited a significant accumulation of ^111^In-Au-HSA NPs in the peritoneal cavity and tumor lesion after IP injection, compared to IV injection. After IP injection, AUC of ascites and tumor were respectively 93- and 20-fold higher, while the AUC of liver and spleen were respectively 12- and 11-fold lower, compared to IV injection. This study demonstrated that Au-HSA NPs are a potential IP drug delivery system in the treatment of peritoneal metastasis. Future goals should be the encapsulation of cytostatic drugs in the Au-HSA NPs to perform in vitro and in vivo anti-cancer efficacy studies.

## 6. Conclusions and future perspectives

Intraperitoneal therapy for PM is a rapidly growing field. Results from recent preclinical and clinical trials have shown a superior efficacy of IP delivery of albumin-bound chemotherapy in the treatment of PM compared to standard chemotherapy formulations. Targeted delivery of chemotherapy is enabled by albumins’ inherent transport properties. Transmesothelial transport of albumin-bound complexes occurs via transcytosis, similar to transendothelial transport. The mechanisms mediating albumin transcytosis in mesothelial cells are not fully elucidated. Therefore, future research should focus on the presence of albumin-binding receptors, mechanisms of albumin transcytosis, and formation of transcytotic vesicles in mesothelial cells. Also, efforts should be made to identify the mechanisms and kinetics of IP albumin-drug dissociation, and to correlate these with pharmacokinetic and pharmacodynamic models, in vivo toxicity, and anti-cancer efficacy. Knowledge of these mechanisms will allow to develop informed designs for further early phase clinical trials using IP albumin-based drug delivery in patients with PM.
